# A quality assurance tool for helical tomotherapy using a step‐wedge phantom and the on‐board MVCT detector

**DOI:** 10.1120/jacmp.v13i1.3585

**Published:** 2012-01-05

**Authors:** Vincent Althof, Paul van Haaren, Rik Westendorp, Tonnis Nuver, Dinant Kramer, Marijke Ikink, Arjen Bel, Andre Minken

**Affiliations:** ^1^ Radiotherapeutic Institute RISO Deventer The Netherlands; ^2^ Department of Radiotherapy, Academic Medical Center University of Amsterdam The Netherlands

**Keywords:** quality assurance, helical tomotherapy, step‐wedge phantom, megavoltage CT detector

## Abstract

The purpose of this study was to develop and evaluate filmless quality assurance (QA) tools for helical tomotherapy by using the signals from the on‐board megavoltage computed tomography (MVCT) detector and applying a dedicated step‐wedge phantom. The step‐wedge phantom is a 15 cm long step‐like aluminum block positioned on the couch. The phantom was moved through the slit beam and MVCT detector signals were analyzed. Two QA procedures were developed, with gantry fixed at 0°: 1) step‐wedge procedure: to check beam energy consistency, field width, laser alignment with respect to the virtual isocenter, couch movement, and couch velocity; and 2) completion procedure: to check the accuracy of a field abutment made by the tomotherapy system after a treatment interruption. The procedures were designed as constancy tool and were validated by measurement of deliberately induced variations and comparison with a reference method. Two Hi‐Art II machines were monitored over a period of three years using the step‐wedge procedures. The data acquisition takes 5 minutes. The analysis is fully automated and results are available directly after acquisition. Couch speed deviations up to 2% were induced. The mean absolute difference between expected and measured couch speed was 0.2% ±0.2% (1 standard deviation SD). Field width was varied around the 10 mm nominal size, between 9.7 and 11.1 mm, in steps of 0.2 mm. Mean difference between the step‐wedge analysis and the reference method was <0.01 mm ±0.03 mm (1 SD). Laser (mis)alignment relative to a reference situation was detected with 0.3 mm precision (1SD). The step‐wedge profile was fitted to a PDD in water. The PDD ratio D20/D10, measured at depths of 20 cm and 10 cm, was used to check beam energy consistency. Beam energy variations were induced. Mean difference between step‐wedge and water PDD ratios was 0.2% ±0.3% (1SD). The completion procedure was able to reveal abutment mismatches with a mean error of ‐0.6 mm ±0.2 mm (1SD). The trending data over a period of three years showed a mean deviation of 0.4% ±0.1% (1 SD) in couch speed. The spread in field width was 0.15 mm (1 SD). The sagittal and transverse lasers showed a variation of 0.5 mm (1 SD). Beam energy varied 1.0% (1 SD). A mean abutment mismatch was found of −0.4 mm ±0.2 mm (1 SD) between interrupted treatments. The on‐board MVCT detector, in combination with the step‐wedge phantom, is a suitable tool for a QA program for helical tomotherapy. The method allowed frequent monitoring of machine behavior for the past three years.

PACS number: 87.55.Qr

## I. INTRODUCTION

Helical tomotherapy Hi·Art system (TomoTherapy Inc., Madison, WI) is an intensity‐modulating radiation technique for cancer therapy.^(^ ^1^ ^,^ ^2^ ^)^ It requires synchrony of gantry rotation, couch translation, linac pulsing, and the opening and closing of the leaves of the binary multileaf collimator (MLC). Furthermore, the system assumes constant output, and a constant size and shape of the longitudinal and transverse profiles. It differs in design and features compared to conventional linear accelerators, which implies that general quality assurance (QA) guidelines are not always applicable or sufficient. The aim of a QA program is to keep actual system properties within tolerances, close to a reference set. The Hi·Art system, which includes hardware and software for the delivery system and dose planning software, is commissioned in the factory to comply with a set of properties, common to all Hi·Art systems. This set is referred to as the Gold Standard beam dataset. The machine should be regularly checked for possible deviations from this dataset and adjusted, if necessary.

A QA program should aim at measuring the performance of isolated items in order to simplify error source tracking. The step‐wedge procedure is designed to do this.

Sarkar et al.^(^ ^3^ ^)^ described tests for several types of binary MLCs. The study focused on MLC design and MLC alignment with other components like linac and collimator. Results from Sarkar's study support the use of the tomotherapy megavoltage CT (MVCT) detector for a number of QA tests.

The effect of variations in output and gantry speed on the delivered dose is discussed by Fenwick et al.^(^ ^4^ ^)^ and Flynn et al.^(^ ^5^ ^)^ The average value of the output obtained during one measurement should still be within 2% while, in case of random angular dose rate variations, effects on delivered dose blur out in helical delivery and a less tight tolerance is allowed.

Balog et al.^(^ ^6^ ^)^ described a QA program which includes megavoltage imaging quality, the accuracy of patient setup correction tools, spatial and temporal accuracy of the dynamic delivery properties, as well as more traditional beam output characteristics. This QA program uses film, water tank, ionization chambers, and the on‐board MVCT detector. It is shown that the transverse beam profile, measured with the on‐board MVCT detector, correlates well with ionization chamber measurements in a water tank. Balog and Soisson^(^ ^7^ ^)^ also presented a QA program for daily, monthly, and annual QA testing. This program includes procedures, specifications, and schedules that are currently implemented in the program recommended by TomoTherapy Inc.

Fenwick et al.^(^ ^8^ ^)^ proposed a QA program that was based on the AAPM‐TG40 conventional linac QA schedule.^(^ ^9^ ^)^ The study provides a detailed overview of 23 relevant parameters to be divided into four categories: static beam dosimetry, system dynamics, system synchrony, and system geometry. Fenwick also proposed a time schedule, consisting of daily, monthly, every three months, and annual checks.

This current study focused on the development of a filmless measurement tool for the relatively short term procedures (daily and monthly) proposed by Fenwick. Only procedures with a static gantry were considered. Some of these procedures are rather labor‐intensive and time‐consuming, especially the ones using film dosimetry. Our purpose was to implement a QA program for tomotherapy machines based on the overview given by Fenwick, but using the MVCT detector to collect attenuation signals and a step‐wedge phantom. With these tools, such a QA program would be extremely time‐efficient. The step‐wedge phantom was provided by TomoTherapy Inc., and was originally developed to monitor beam energy using an ionization chamber mounted on an in‐air scanner. The development of our QA procedures, including the algorithms to analyze the data, was performed independently and before development of the TQA application of TomoTherapy Inc. Most of the functionality discussed in this paper is currently available in the tomotherapy product. In extension to what TQA offers, this article describes and validates the completion procedure. It checks the abutment of an interrupted and resumed treatment. This paper is an independent confirmation and verification on the use of the tomotherapy MVCT detector for QA procedures. These procedures were designed as constancy measurement tools (i.e., to detect a deviation from a reference). The QA tools have been in use since August 2007. Trend information of machine behavior since then will be presented.

## II. MATERIALS AND METHODS

Two QA procedures were developed: 1) step‐wedge: to check alignment of the lasers and the virtual isocenter, couch movement and velocity, beam energy consistency, and field width; and 2) completion: to check the accuracy of the abutment of treatment fields for a completion made by the tomotherapy system after a treatment interruption.

**Figure 1:**
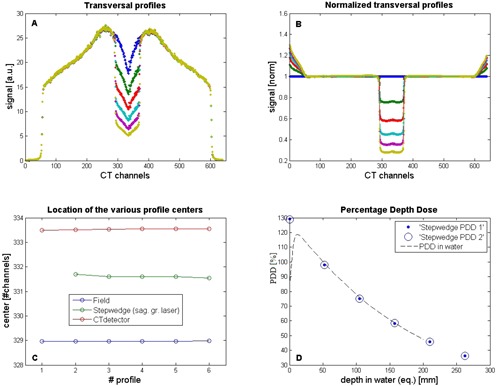
Analyses of the transverse profiles of the MVCT detector during the step‐wedge QA procedure: (A) profiles over all MVCT detector channels measured for the five separate steps, given in arbitrary units (a.u.), and used for center of field determination; (B) MVCT detector profiles (panel A) normalized to the profile measured in air, used to measure the position of the sagittal laser; (C) location of the center with three different methods; (D) percentage depth dose (PDD) derived from the six levels of the non‐normalized MVCT profiles (‘step wedge PDD 1’), and derived from the normalized MVCT profiles (‘stepwedge PDD 2’), normalized to the dose value at 5 cm water depth.

A common aspect of both procedures is the use of the on‐board MVCT detector which opposes the linear accelerator and measures the transmitted radiation. This detector consists of 640 xenon filled cavities, separated by tungsten septa. Each cavity has a physical width in lateral direction of 1.2 mm and a length in longitudinal direction of 42 mm, corresponding to a width of ≈0.7 mm and a length of≈25 mm, back projected at isocenter level. The source‐to‐axis distance (SAD) of a tomotherapy machine is 85 cm, the source‐to‐detector distance (SDD) is 142 cm in the center of the MVCT detector, and the radius of curvature of the MVCT is 110 cm. Because of this difference in MVCT curvature and SAD, ray lines emanating from the focus are bisecting the septa walls, except on the beam axis. Therefore, an increased signal is measured for the off‐axis MVCT detector cells caused by scattered photons, and a typical dip is seen in the profile center for the on‐axis cells (e.g. Fig. 1(A)).

The tomotherapy system stores the detector and machine data while irradiating. The detector data file consists of readout signals of the 640 MVCT channels and 85 system signals, such as monitor chamber signals (used to correct for output variations), water temperature, water pressure and waterflow, air pressure, jaw settings, couch and gantry position. The data were extracted from the system through an FTP connection. All system signals were acquired at a frequency of 300 Hz and were down‐sampled to 30 Hz by averaging 10:1. This speeds up the process of data transport and analysis. The data files were analyzed and visualized with in‐house developed tools (Delphi, MATLAB (The MathWorks, Natick, MA)). This study concentrates on the MVCT detector signals acquired with the step‐wedge phantom in the beam. The acquisition (beam on) time of the step‐wedge procedure is 300 sec.

### A. Step‐wedge procedure

**Figure 2:**
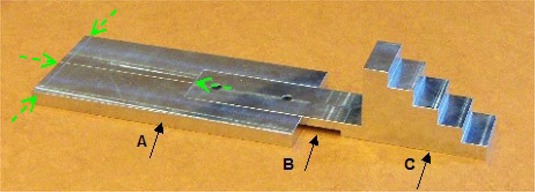
Step‐wedge phantom: (A) support for alignment to the lasers; (B) notch for couch end; (C) step wedge. The dashed arrows show the engraved lines for laser setup.

The step‐wedge phantom is staircase‐like, and is attached to a rectangular support for alignment to the lasers with a notch to position the wedge on the couch such that it extends beyond the top of the couch (see Fig. 2). If the couch type ‘High Performance’ is used, the phantom is positioned simply on the couch top. The phantom is set up on the couch such that the sagittal and transverse laser lines match the grooves carved on the phantom. The step wedge is 150 mm long and 69.7 mm wide and consists of five steps with lengths of 29.9, 30.0, 30.0, 30.0, and 30.1 mm respectively. The thicknesses of the steps are 19.5, to 39.0, 58.5, 78.0, and 97.6 mm, respectively. The step wedge is made of massive aluminum 6061, with a density of 2.69 $\text{gr}/{\text{cm}}^{3}$, according to the manufacturer (TomoTherapy Inc.). This corresponds to water equivalent steps of 52.5, 104.9, 157.4, 209.8 and 262.5 mm, respectively.

The step‐wedge procedure is performed with a static gantry at 0°, a nominal field width of 1 cm, and with all leaves open, while couch and step wedge move 20 cm into the gantry. To be able to detect the transition from one step‐wedge level to the next, the longitudinal field size must be smaller than the longitudinal length of the CT detector channels (and also smaller than the longitudinal length of the step‐wedge levels). Therefore, this procedure is only suitable to be performed with the 1 cm field size. This is a minor limitation, because a drift in one of the parameters measured with the 1 cm field will also show up for the other field sizes, either because the parameter is independent of field size (laser position, couch speed, completion) or the parameter will be affected also for the other field sizes (energy).

Only one set of collimator encoders is used for all field sizes. As a result, a drift in encoder value will have the biggest relative effect for the small 1 cm slit. So, if the 1 cm slit size is within tolerance, the bigger field sizes will be as well.

#### A.1 Step‐wedge procedure: time profile analysis

**Figure 3:**
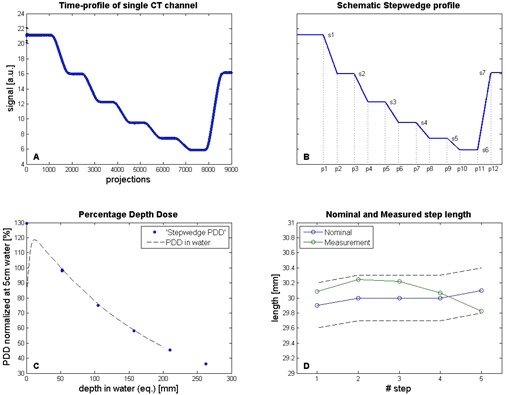
Analyses of the time profile of one MVCT detector channel during the step‐wedge QA procedure: (A) MVCT detector signal course over time for one CT channel, corrected for output (monitor chamber) given in arbitrary units (a.u.); (B) schematic profile fitted to the MVCT signal time course (panel A); (C) percentage depth dose (PDD) derived from the six fitted step‐wedge profile levels, normalized to the dose value at 5 cm water depth; (D) measured length of the five separate steps (green symbols) derived from the fitted step‐wedge profile compared with the nominal step length (blue symbols) within a range of ±1% (dotted lines). Measured points are connected with solid lines to enhance visibility.

All signals from the MVCT detector channels were corrected for fluctuations in the machine output as measured with the monitor chamber of the machine. The time profile analysis was performed using the signal from one centric CT channel (Fig. 3(A)) of the MVCT detector array. The delivery was performed in 300 sec using a sampling frequency of 30 Hz. This corresponds to 9000 obtained samples (‘projections’).

To enable an accurate analysis of the obtained signal, a model of the expected signal was made (Fig. 3(B)). The schematic step‐wedge profile consists of seven flat and six sloped straight lines, and is characterized by the 19 parameters, p1–p12 and s1–s7. Parameters p1–p12 correspond to the projection numbers of the transitions between flat and sloped lines in the profile. Parameters s1–s7 correspond to the signal levels of the subsequent flat lines, representing the measured signal levels for the different step thicknesses of the step wedge. The first level is measured in air with the step wedge not yet in the irradiation beam.

A fit procedure was used to transform the schematic step‐wedge profile to the measured signal by minimizing the summed square of differences. Using an unconstrained nonlinear optimization technique (*fminsearch*, MATLAB),^(^ ^10^ ^)^ the fit yields an accurate estimate of the optimized parameters, p1–p12 and s1–s7.

Initially, to test the robustness of the fitting procedure, the offset of the points was chosen randomly 10 times, resulting in p1 to lie in the interval of projection 1150–1250.

The expected value of p1 is 1200. For each start position, the ‘least squares’ fit is performed and the best fit out of 10 is chosen. The result is a schematic step‐wedge profile which represents the measured profile. The resulting fit has a high quality ($R^{2}>0.99$). Therefore, the exact positions of the transition points between flat and sloped lines in the modeled profile are an adequate and accurate presentation of the edges in the measured time profile. Analysis of the profile (i.e., the position of the transition points) is reduced to analysis of a set of flat and sloped lines. Examples of measurements and analyses from the step‐wedge procedure are shown in Figs. 3 and 1.

##### A.1.1 Step‐wedge procedure time profile: energy

The thicknesses of the five step‐wedge levels were converted to equivalent depths in water, using the material's density. A function with an exponential decay is used to fit signal levels s1–s7, again using the unconstrained nonlinear optimization technique (*fminsearch*, MATLAB). From this fit a percentage depth dose (PDD) is obtained (Fig. 3(C)), which is normalized at 5 cm water depth. The PDD values at 10 and 20 cm were read out and the ratio D20/D10 was calculated. This ratio was compared to the ratio calculated from the PDD measured in a water phantom during commissioning of the machine. This PDD was also normalized at 5 cm water depth (Fig. 3(C)). The ratio can be used to check beam energy/spectrum consistency as an alternative to the quality index.

##### A.1.2 Step‐wedge procedure time profile: couch velocity

Values for parameters p1–p12 (in ‘projections’, which is actually ‘time’) were obtained from the fitted schematic step‐wedge profile, and were used to check velocity consistency of the treatment couch and to measure the field width in longitudinal direction (i.e., the direction of couch movement).

Multiplying the numbers of projections between transitions (p1–p3, p3–p5, etc.) by the time per projection (30 msec), gives the time per level. The apparent length of each level is calculated by multiplying the time per level by the nominal couch speed. Comparison of the lengths obtained from this analysis with the physical lengths of the steps for all steps together and for the individual steps is a check on couch velocity, as well as uniformity of the couch velocity. Measured and nominal step lengths are plotted in Fig. 3(D), and should be within ±1%.

##### A.1.3 Step‐wedge procedure time profile: field width

The calculation of the field width of the irradiation beam is based on the number of projections between transition p2–p1, which corresponds to the first step of the step wedge. The field width is calculated by multiplying the time of projection p2–p1 by the couch speed. This value is referenced to an actual measurement of the field width using a reference method (in this case a measurement of the longitudinal profile in solid water).

##### A.1.4 Step‐wedge procedure time profile: transverse laser position

Additional analysis from the time profile includes a check of the position of the transverse laser. Since the step wedge has been aligned to the lasers, which should be in the virtual isocenter, its exact longitudinal position was retrieved from the first transition in the profile: p1 in projections. This transition represents the moment the step wedge enters the irradiation beam. The projections are converted to position by multiplying with the ‘time per projection’ and the couch speed. This position is compared with a reference position.

#### A.2 Step‐wedge procedure: transverse profile analysis

Transverse profiles were constructed from the signals of all 640 MVCT detector channels when the beam in longitudinal direction completely projects over the flat parts of the step wedge. Figure 1(A) shows transverse profiles of all five step‐wedge levels and one profile through air. All transverse profiles were normalized to the profile measured in air, in order to extract the influence of the step wedge (Fig. 1(B)).

The central part of both non‐normalized and normalized transverse profiles was described with so‐called modified Gaussian‐shaped curves:

(1) $$y_{fit}=y_{0}+a\cdot \text{exp}\left(-\frac{1}{2}{\left(\frac{\left|x_{meas}-x_{0}\right|}{b}\right)}^{c}\right)$$

with $y_{\mathrm{fit}}$ the values of the dependent variable (here CT signal) calculated for all values $x_{\mathrm{meas}}$ of the independent variable (here CT channel). Fit parameters $y_{0},a,x_{0},b,$, and *c* represent offset, amplitude, center, width, and shape of the modified Gaussian model peak, respectively.

A fit was performed (fminsearch) to transform this schematic step‐wedge profile to the measured profile. Fit‐parameters were used to determine the position of the center of the profiles, resulting in accurate, robust, and objective analyses. These positions could not be determined accurately enough directly from the measured profiles, due to noise in the measurements. The fit procedure assumes a symmetric profile, which is the case when the phantom is positioned close to the center of the slit beam. A small asymmetry can be seen in the transverse profile in Fig. 1(A). This asymmetry was also seen in the profile measured in the water tank and is, therefore, a property of the profile itself. It did not influence the fit in a notable way. The quality of the fits was very high ($R^{2}>0.99$).

##### A.2.1 Step‐wedge procedure transverse profile: sagittal laser position

The procedure is used for determination of three center positions. First, from the value of $x_{0}$ for the non‐normalized profile (Fig. 1(A)), the position of the typical dip in the transverse detector profile is found. This is the CT channel for which the ray lines originating from the focus bisect the septa walls of the detector at its minimum. This channel opposes the focus and is called the center channel of the MVCT detector.

Secondly, the values of $x_{0}$ that result from the fit of the normalized profiles (Fig. 1(B)) represent the exact sagittal position of the step wedge. This corresponds to the position of the sagittal laser, since the step wedge was aligned to the lasers.

The third ‘center’ found in this procedure is the center of the irradiation field. This is not determined from a fit, but from the second derivative of the left and right flanks of the non‐normalized profiles. At the exact position of the flanks, the second derivative is equal to zero.

If the system (linac, mlc, and detector) is aligned perfectly, all three centers coincide. The position of the sagittal laser should coincide with the center of the irradiation beam (i.e., with the isocenter of the treatment machine). An example of an analysis result is shown in Fig. 1(C). All three positions are depicted in this figure. In this specific case, the sagittal laser was approximately 2.6 CT channels misaligned with the center of the irradiation beam, which corresponds to a deviation of 1.9 mm at isocenter level. This was outside the tolerance level, and the laser was adjusted accordingly.

If the center position of the step wedge, calculated for all five steps of the step wedge, is not constant, the laser and/or the direction of the couch movement do not coincide with an axis perpendicular to the MVCT imaging plane. Such a mismatch does not affect the amount of step‐wedge material traversed by the beam, and thus will have no effect on the PDD.

Similar to the method described above for the time profile, a PDD can be constructed from the (fitted) transverse profiles for the different steps of the step wedge (Fig. 1(D)). We can use the fit data from the non‐normalized transverse profiles (closed symbols), or the fit data from the normalized profiles (open symbols) (see Fig. 3(C)). Both are in good agreement. The analysis of the PDD is the same as described earlier in Section A.1.1, ‘Step‐wedge procedure time profile: energy’ above.

#### B. Completion procedure

In addition to the tests proposed by Fenwick et al.^(^ ^8^ ^)^ we implemented a procedure to check the ‘completion’ (i.e., the abutment of the treatment fields before and after an interruption). Such an interruption may happen during treatment of a patient. The tomotherapy system automatically generates a new procedure based on what remained of the treatment. An accurate abutment is necessary to prevent under‐ or overdosage in the abutment region.

**Figure 4:**
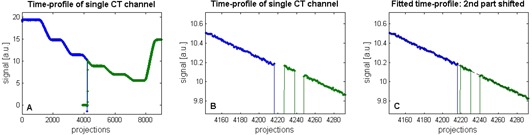
Analysis of a completion procedure (profile intensities are given in arbitrary units (a.u.)): (A) time profiles of the step wedge from both procedures; (B) detail of panel A (the square shown in panel A) at the junction of both time profiles, a small mismatch can be seen, and the dip in signal around projection 4240 is a property of the newly‐generated treatment procedure; (C) the mismatch has been corrected by translation of the second profile to the left until the profile is exactly in line with the first profile. The shift performed is eight projections, which corresponds to 0.2 mm.

To perform a completion procedure, a step‐wedge procedure is interrupted during radiation at the point when the time profile of the step wedge shows a slope in between two flat parts. The new procedure is selected from the tomotherapy database and the remainder of the treatment can be delivered. The position of the step wedge on the couch top is not changed. To deliver the remaining part of the procedure, the couch is retracted first to the reference position such that the step wedge matches the transverse laser line again. In the analysis, the detector data files of both procedures were combined, and the translation correction needed for a perfect abutment of both step wedge time profiles is obtained in number of projections and converted to mm using ‘time per projection’ and couch speed. Since the completion procedure has a fixed gantry angle of 0° and all leaves are open, it only checks if the couch position is correct at the resumption of treatment delivery. This is not trivial because the couch has to move into the bore to the correct start position and has to travel at constant speed at the moment the beam is on and the leaves are opened. An example of the completion procedure data analysis is shown in Fig. 4.

#### C. Verification methods

The ability of the step‐wedge method to detect and quantify variations in beam energy, field width, couch speed, and laser position was measured and compared with a reference method.

Beam energy was varied by varying the gun injector current (Injector I) between 3.1 and 5.0 Vdc. This was done on a nonclinical machine. Normal operating Injector I values lie between 3.5 and 4.5 Vdc. The ratio of the dose measured at depths of 20 and 10 cm (D20/D10) is a measure for beam energy. The measurement using step wedge and MVCT detector was compared with a measurement using solid water slabs and ionization chambers. The latter was considered the reference method. The solid water was placed on top of the couch and was kept at a constant position in the beam during these measurements. The measurement in solid water was done with two calibrated A1SL chambers at 10 and 20 cm depth, simultaneously. Two TANDEM electrometers from PTW (Freiburg, Germany) were used to collect charge. No correction was made for chamber shift. The reading was corrected for temperature and pressure and converted to dose with proper calibration factors. The ratio D20/D10 was varied 6% between 0.502 and 0.535.

Field width and couch speed were varied by changing corresponding parameters in the tomotherapy procedure application. The field width measured with the step‐wedge procedure was compared with a measurement performed with an ionization chamber in solid water. The ionization chamber was oriented with its long axis perpendicular to the couch movement. It is positioned in the center of the beam and at depth $d_{\max}=1.5\text{cm}$. The surface of the solid water was at isocenter height. The position of the ionization chamber was checked with an MVCT and corrected if necessary. Field size was defined at FWHM of the beam profile and scaled to isocenter. The ionization chamber method was considered the reference method. Note that both methods assume a constant and known couch speed. The couch was driven through the radiation field with a constant speed of 1 mm/sec, which was the same speed as used for the step‐wedge procedure. The chamber electrometer sample rate was 0.2 sec. The gantry was kept stationary at 0°. The field width of the nominal 10 mm slit was varied between 9.7 mm and 11.1 mm in steps of 0.2 mm by varying the encoder values of the back jaw and front jaw of the tomotherapy collimator system.

It was checked if the step‐wedge procedure was capable of detecting different velocities of the couch table top, induced by changing the appropriate parameter in the tomotherapy procedure application. The different speeds inputted in the tomotherapy application served as the speeds ‘that should be measured’. The couch speed was varied in steps of 0.5% between −2% and +2% from the default value of 1 mm/sec.

A deviation of the sagittal and longitudinal laser to the calibrated position was simulated by varying lateral and longitudinal position of the step wedge in the beam. The couch drive mechanism was used to achieve this. The accuracy of couch positioning was checked with a micrometer dial gauge and was accurate within 0.1 mm. For the sagittal laser, the left‐right position of the step wedge was varied between −3mm and +3mm, in steps of 1 mm. For the lateral laser, the in‐out position of the step wedge was varied between −3mm and +3mm.

To check the accuracy of the completion method, an experiment was set up to simultaneously measure the abutment with film (EDR2) and with step wedge. A film was placed on the couch and the step wedge was placed on top of the film. The film was processed in an AGFA CP1000 film processor. Mismatches were introduced by small longitudinal translations (−1.0, −0.5, 0, 0.5, and 1.0 mm) of the couch when the system was set up for the remaining part of the treatment.

#### D. Monitoring machine behavior

The step‐wedge procedure has been in use since August 2007. The analysis results are stored in a database. Trending information measured with the step‐wedge procedures, obtained over a period of three years, is presented.

### III. RESULTS

#### A.1 Step‐wedge procedure: energy

Table 1 shows the ratio D20/D10 measured at depths of 20 cm and 10 cm (equivalent depth in water). This ratio is derived from measurements with ionization chambers placed in solid water (reference method, 2nd column), and from measurements with the step wedge (3rd column). The percentage difference in ratio between solid water and step wedge is shown in the 4th column of Table 1. Overall, the ratio obtained with the step wedge followed the ratio measured using the ionization chambers with a mean difference of −0.2%.

**Table 1:** Accuracy of the energy determination by the step‐wedge procedure. Changes in beam energy were invoked by changing the Injector I. The ratio D20/D10 is measured using ionization chambers in solid water and using the signals registered by the MVCT detector for the different step‐wedge levels.

*Injector I (Vdc)*	*Solid Water*	*D20/D10 Step Wedge*	*Difference (%)*
3.10	0.535	0.536	0.2
3.96	0.521	0.520	−0.2
4.07	0.519	0.519	0.0
4.26	0.516	0.514	−0.4
5.00	0.502	0.499	−0.6

#### A.2 Step‐wedge procedure: couch speed

**Figure 5:**
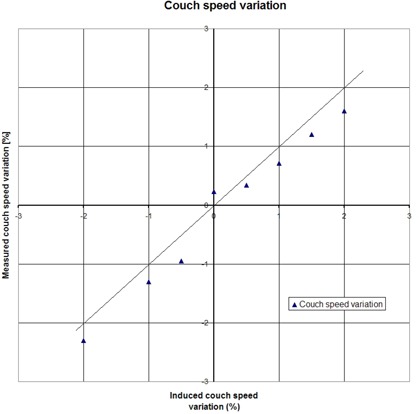
Accuracy of the step‐wedge procedure: couch speed. Couch speed is varied by changing the appropriate parameters in a tomotherapy treatment procedure. The actual couch speed is obtained by analysis of the time profile of the step‐wedge phantom, measured with the MVCT detector.

The horizontal axis of Fig. 5 shows the induced couch speed variation. The vertical axis shows the couch speed variation measured using the step‐wedge procedure. There is a slight systematic underestimation of the measured couch speed of about 0.17% (mean absolute difference) with a variation of 0.2% (1 SD).

#### A.3 Step‐wedge procedure: field width

**Figure 6:**
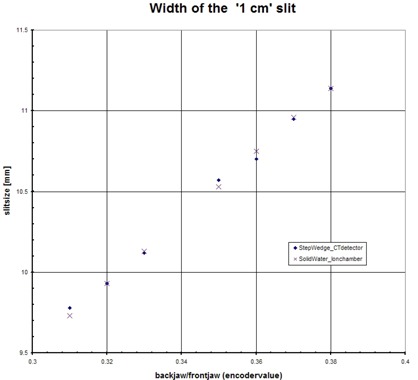
Accuracy of the step‐wedge procedure: field width. Field width in longitudinal direction is measured with an ion chamber in solid water and by analysis of the time profile of the step‐wedge phantom, measured with the MVCT detector.

The accuracy to measure changes in field width is shown in Fig. 6. The resulting field widths were measured using an ionization chamber in solid water (reference method) and using the step‐wedge procedure. Both measurement methods were in good agreement with a mean difference < 0.01 mm and a variation of 0.03 mm (1SD). The step‐wedge method is well‐suited to measure changes in field width.

#### A.4 Step‐wedge procedure: laser position

**Figure 7:**
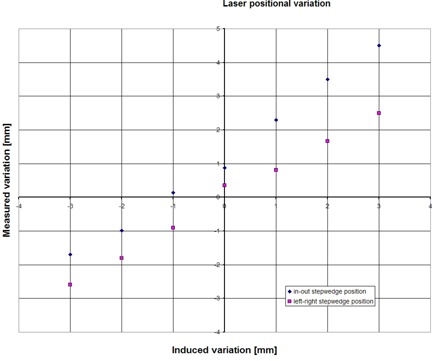
Accuracy of the step‐wedge procedure: variation in laser position. Variation in laser position is simulated by moving the couch (and the step wedge on top of it) a certain amount. Shown are the ability to measure the induced variation by analysis of the time profile of the step‐wedge phantom, measured with the MVCT detector, for the lateral laser (in–out position) and the sagittal laser (left–right position).The in–out offset can be eliminated by proper calibration of the couch starting position.

Figure 7 shows the precision of the detection of the sagittal (left–right) and lateral (in–out) laser position. Sagittal laser (mis)alignment was detected with 0.3 mm precision (1SD). The detection of a variation in in–out position showed an offset of 1.2 mm, with a standard deviation of 0.4 mm. This offset is constant, but can be corrected for by proper calibration of the procedure.

### B. Completion procedure

Figure 4(A) shows the overall step‐wedge time profiles of both procedures, without correction of an abutment error. The flat part at the beginning of the second profile corresponds to 10 seconds ‘beam on’ with closed MLC. This allows the system to stabilize output and couch speed. Figure 4(B) zooms in on the profiles in the small square (visible in Fig. 4(A)). This reveals a small mismatch which has been corrected in Fig. 4(C) by a shift to the left over eight projections, which corresponds to 0.2 mm. A second dip in the time profile is visible. This is caused by the closing of all leaves for a few msec. The reason why the sinogram (leaf open time per leaf per projection) is designed to show this behavior, is not clear to us.

The abutment of two longitudinal profiles is simulated using the known penumbra values of the 1 cm slit. A shift of 1 mm in the abutment will cause a peak or dip in dose of about 8%.

The film, positioned under the step‐wedge and irradiated simultaneously during the completion procedure, was analyzed in VeriSoft, a film analysis package from PTW (Freiburg, Germany). The mean optical density in the abutment region was 0.8. A line profile was taken perpendicular to the abutment and was analyzed in a spreadsheet.

With film slightly lower peak and dip maxima were found: 6% for the 1 mm gap/overlap and 3% for the 0.5 mm gap/overlap. Although it was not trivial to relate the height of the peak or dip with a mismatch in mm because of the presence of film processing artifacts, the film measurement was able to reveal a mismatch of 0.5 mm.

The simultaneously‐performed completion procedure with the step wedge showed a gap of −0.6 mm, with a variation of 0.2 mm (1 SD). Despite of this gap, the spread was small and the step‐wedge results closely followed the induced mismatches.

### C. Monitoring machine behavior

**Figure 8:**
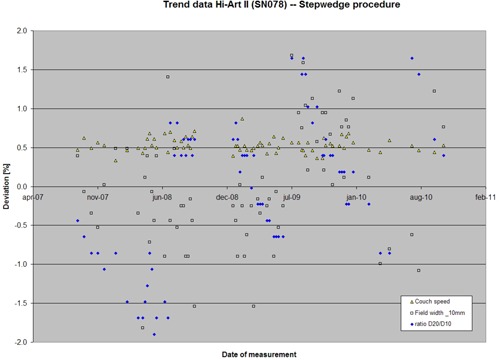
Machine behavior over a three‐year period. Behavior is measured with the step‐wedge procedure. Shown are variations in couch speed (nominal 1 mm/sec), field width (nominal 10 mm at iso), and ratio D20/D10. Deviations are presented in percentages, relative to a reference value. The jumps in D20/D10 ratio correspond to subsequent target changes.

The second column in Table 2 shows the variation in analysis with the step‐wedge procedure repeated 10 times on the same dataset. The third column in Table 2 shows the machine behavior for our two Hi•Art II machines. Because the machines showed similar behavior, the data is averaged over both machines. The trending data was collected between August 2007 and October 2010. Trending data of one of the machines are shown in graphical form in Fig. 8.

**Table 2:** Reproducibility of the step‐wedge method by repeated analysis on same dataset (10 times) and trending behavior of our two Hi•Art II machines (averaged) during a three‐year period (August 2007 to October 2010). Number of measurements in this period was 65 per machine.

*Item*	*Reproducibility (1SD)*	*Trending Data (1SD)*
Couch speed overall dev (%)	0.03	0.1 (mean 0.4)
Couch speed level dev (%)	0.14	0.2
Energy D20/D10 (%)	<0.1	1.0
Laser transverse (mm)	0.03	0.4
Laser sagittal (mm)	<0.01	0.6
Field width (mm)	0.04	0.15
Abutment mismatch (mm)	0.1	0.2 (mean −0.4)

## IV. DISCUSSION

A quality assurance (QA) program for helical tomotherapy differs from a QA program for conventional linear accelerators because of its unique design (i.e., the binary MLC, couch movement and helical delivery). We have developed two QA procedures which focus on the delivery system, using the on‐board MVCT detector, the step‐wedge phantom and system parameters recorded during radiation. The approach is time efficient. It allows the measurement of multiple items in one procedure, with a fully‐automated analysis. The whole procedure takes about 15 min, whereas measurement of the same QA items with conventional methods (ionization chamber and film) would take about 90 min. The QA items involved should be measured on a weekly and monthly basis as described by Fenwick et al.^(^ ^8^ ^)^ Measurement of the coronal laser and output are not part of this step‐wedge procedure and will have to be measured separately.

Target wear appears to be more rapid on the Hi•Art II machine than on a conventional linac. Therefore, the energy of the treatment beam will change during target life.^(^ ^11^ ^)^ In accordance with TG142,^(^ ^12^ ^)^ the tolerance for beam quality variations is 1%. The step‐wedge procedure allows an easy beam energy consistency measurement with an accuracy within 0.5% (relative to the reference method, Table 1). A slightly greater difference of −0.6% was found at the largest ratio D20/D10, but this ratio was reached by tuning the Injector I beyond the operating limit of 4.50 Vdc and is, as such, not a clinical situation. It was observed that the CT detector response has some energy dependence, but the effect on the ratio PDD 20/10 will be limited. The step‐wedge phantom will be equipped with a hole to insert an ionization chamber. This will be used as output measurement which can be performed during the same procedure. This is not implemented in the current step‐wedge phantom.

Trend data for a period of three years shows a variation in D20/D10 of 1.0% (1 SD). It should be noted that this data comprises the history of a number of target changes. Due to target wear, the energy drifts slowly to lower values, which subsequently can be corrected by tuning the Injector I. As can be seen from the trending data in Fig. 8, we have been conservative in tuning the energy. Changes up to 2.5% were allowed, as long as results of phantom measurements with ionization chambers kept within 3% on average. This change in energy appears to be a predictor of target failure. This enables one to schedule target replacement in preventive maintenance.

The constancy of the longitudinal profile shape and size is particularly important for helical tomotherapy. The dose to the patient is the integration of the longitudinal profile with couch motion.^(^ ^13^ ^)^ Typically, the dose delivered will change approximately ±2% if the nominal 10 mm field width changes by 0.2 mm. The step‐wedge procedure allows fast and accurate measurement of changes in field width. Trend data for a period of three years shows a variation in field width of 0.15 mm (1 SD).

The position of the transverse laser (in–out position) and the sagittal laser (left–right position) can be accurately measured using the step‐wedge method. It should be noted that an incorrect position of the lasers will not result in an incorrect patient treatment if online 3D setup verification and correction is performed using the build in MVCT detector.

Theoretically, also the position of the coronal laser (the height of the couch) can be checked with the step‐wedge procedure by analyzing the width of the transverse profiles. However, this measurement was not sensitive enough to deviations in laser position (> 1 mm). To measure the position of the coronal laser, it is better to operate the step‐wedge procedure with a gantry angle of 90° or 270° or in helical mode.

The tomotherapy control system allows online monitoring of machine parameters with a sample rate of 300 Hz. An interrupted treatment, therefore, can be continued with high abutment accuracy. This is especially important because of the sequential nature of the tomotherapy dose delivery. The completion procedure presented in this study is time‐efficient, and is able to detect abutment errors of 0.5 mm. This will reveal under‐ or overdosage of 3% at the abutment region (based on film measurements). An advantage of the step‐wedge analysis is the unambiguous presentation of the mismatch in mm. The completion procedure described here is performed with the gantry fixed at zero degrees. The clinical value in performing such a limited test is that this procedure tests the ability of the system to start the remainder of the treatment at the correct couch position and with the correct synchronization of MLC and couch. These parameters are also crucial for a correct treatment.

In clinical practice, the accuracy of the abutment will also be influenced by correct positioning of the patient on the couch. To verify this, 3D patient setup verification should be performed with the on‐board megavoltage CT detector just before the delivery of the remainder of the treatment.

In the Hi•Art II system, couch movement is not synchronized with gantry movement and MLC dynamics. Careful and frequent control of couch speed and couch speed uniformity is therefore important. Trending data shows the variation in couch speed during three years is limited (mean 0.4%, 0.1% 1SD) and within tolerances (1%).

While synchronization of couch with gantry and couch with MLC is not controlled by the system, it is important to have a tool to measure and analyze the synchronicity of these items. To achieve this, the step wedge should be adapted for use in helical mode. This will also allow one to analyze the completion procedure in a more comprehensive way — not only the abutment of fields based on correct couch positioning, but also based on correct gantry position and leaf motion.

The step‐wedge method showed reproducible results in repeated analysis on the same dataset. The variations were small relative to the variations seen in the trend data (Table 2). This implies the (fit) methods used to analyze the measured detector data are robust and that the step‐wedge procedure is an adequate tool to perform constancy measurements. Our measurements show the step‐wedge method accurately measures induced changes in beam energy, field width, couch speed, laser position, and abutment of treatments. Over a period of three years, our two Hi•Art II systems have been monitored extensively using the new methods presented in this study.

## V. CONCLUSIONS

This study shows the suitability of a step‐wedge phantom and the on‐board MVCT detector of the tomotherapy Hi•Art for QA purposes. A comprehensive set of QA items can be measured accurately in a quick, simple, and automated way. The new method allowed frequent monitoring of machine behavior for the past three years.

## ACKNOWLEDGMENTS

TomoTherapy Inc. is acknowledged for providing the step‐wedge phantom.
